# Improved eye- and skin-color prediction based on 8 SNPs

**DOI:** 10.3325/cmj.2013.54.248

**Published:** 2013-06

**Authors:** Katie L. Hart, Shey L. Kimura, Vladimir Mushailov, Zoran M. Budimlija, Mechthild Prinz, Elisa Wurmbach

**Affiliations:** 1 Formerly at the Office of Chief Medical Examiner of the City of New York, Department of Forensic Biology, New York, NY, USA; 2 Office of Chief Medical Examiner of the City of New York, Department of Forensic Biology, New York, NY, USA; The first two authors contributed equally to this study.

## Abstract

**Aim:**

To improve the 7-plex system to predict eye and skin color by increasing precision and detailed phenotypic descriptions.

**Methods:**

Analysis of an eighth single nucleotide polymorphism (SNP), rs12896399 (*SLC24A4*), showed a statistically significant association with human eye color (*P* = 0.007) but a rather poor strength of agreement (κ = 0.063). This SNP was added to the 7-plex system (rs12913832 at *HERC2*, rs1545397 at *OCA2*, rs16891982 at *SLC45A2*, rs1426654 at *SLC24A5*, rs885479 at *MC1R*, rs6119471 at *ASIP*, and rs12203592 at *IRF4*). Further, the instruction guidelines on the interpretation of genotypes were changed to create a new 8-plex system. This was based on the analysis of an 803-sample training set of various populations. The newly developed 8-plex system can predict the eye colors brown, green, and blue, and skin colors light, not dark, and not light. It is superior to the 7-plex system with its additional ability to predict blue eye and light skin color.

**Results:**

The 8-plex system was tested on an additional 212 samples, the test set. Analysis showed that the number of positive descriptions for eye colors as being brown, green, or blue increased significantly (*P* = 6.98e-15, z-score: -7.786). The error rate for eye-color prediction was low, at approximately 5%, while the skin color prediction showed no error in the test set (1% in training set).

**Conclusions:**

We can conclude that the new 8-plex system for the prediction of eye and skin color substantially enhances its former version.

Recording the visible characteristics of a decedent holds significant forensic importance because corroboration of these traits with missing persons reports may lead to identification. Forensic anthropologists can determine some of these characteristics, such as height, sex, and ethnicity, but pigment-related features may not be inferred after remains have become decomposed. In these cases, DNA can be a vital forensic tool.

Genetic variances such as single nucleotide polymorphisms (SNPs) can provide some information about phenotypic characteristics of individuals, including pigmentation of the iris, hair, and skin (1-4). Pigmentation is a complex trait that depends on genetics as well as on other factors, including environmental factors and certain drugs (5,6). Melanin is the main pigment of the iris, hair, and skin. It is packaged in specialized subcellular compartments, melanosomes, which are exported to adjacent keratinocytes where most pigment is found (7). Differences in pigmentation arise from variations in number, size, composition, and distribution of melanosomes (8) and account for the diverse combinations of iris, hair, and skin coloration that can be observed among individuals around the world (9).

Genome-wide association studies (GWAS) pointed to certain genes, regions, and SNPs that demonstrated a relevance to pigmentation (10-16). SNPs that were found to correlate with eye, hair, or skin color were analyzed further to determine if they could be meaningful for incorporation into a DNA test to predict individual phenotypes for these traits (17-19). A predictor, as defined here, includes a selection of well-defined SNPs and unambiguous instructions to interpret genotypes, ensuring a standardized procedure.

With many candidate SNPs available, several predictors have been published for the evaluation of pigmentation (17,18,20-23). One of the systems, “IrisPlex,” determines an individual’s eye color and, following expansion to “HIrisPlex,” hair color (18,19). “HIrisPlex” utilizes 24 genetic variants from 11 genes (*MC1R*, *TYR*, *EXOC2*, *SLC45A2*, *TYRP1*, *SLC24A4*, *KITLG*, *ASIP*, *HERC2*, *OCA2*, and *IRF4*). Twenty-two of these variants are used to determine hair color and six eye color with four used for both predictors (19).

Another system is based on seven SNPs from seven genes (*HERC2, OCA2, SLC45A2, SLC24A5, MC1R, ASIP,* and *IRF4*), predicting eye and skin color. Six of the seven SNPs are used to predict eye color and all seven for skin coloration. The 7-plex system follows a decision tree method, starting with rs12913832 which leads to a “negative” description, not blue or not brown. By including genotypes of five additional SNPs, the results can lead to a “positive” description for brown or green eye colors. The prediction of skin color follows a similar process of elimination that distinguishes between skin that is not dark or not light using only homozygous genotypes (17). The 7-plex system was tested on 554 samples of various populations and validated on 251 additional samples (17,24). Validation revealed low error rates: 3% for the eye-color and 1% for the skin-color prediction (24). Call rate for eye-color prediction was 100%. Call rate for skin-color prediction varied from 5%-85% depending on the population (24).

In this study, the 7-plex system was further developed. The inclusion of an eighth SNP and changes in the instructions for interpreting data were developed on a training set of 803 samples and yielded in more detailed, positive descriptions of all eye color bins (ie, blue, green, and brown) as well as one skin color bin (light). The developed 8-plex system was tested on 212 additional samples, the test-set, which was newly-collected.

## Materials and methods

### Sample collection and data acquisition

Before donating a sample, each volunteer read and signed the consent form. 1015 samples (803 training set and 212 test set) from non-related individuals were collected. This project was approved by the New York City Department of Health and Mental Hygiene Institutional Review Board that serves as Institutional Review Board (IRB) for the Office of Chief Medical Examiner (OCME) (IRB# 08-066). Sample collection at New York University (NYU) was approved by their IRB (H#:09-0739).

For precise data acquisition, each volunteer filled out a questionnaire which asked for detailed information on eye, hair, and skin coloration. Participants were also asked for the geographic population with which they most associate. Pictures, under defined conditions, were taken for confirmation. Skin color was evaluated from the inner side of the upper arm, since this area is not routinely exposed to sunlight.

### Sample binning

Collected eye and skin color information was assigned into three bins: blue, green, or brown for eye color; and light, medium, or dark for skin coloration. The populations were distinguished among African-American, South Asian, East Asian, European descendants, and mixed, which included Hispanics (25) as well as individuals whose parents were not associated with the same geographic population.

### DNA extraction

Buccal swabbing was used to collect DNA samples. DNA extractions were performed following the instructions of the manufacturer (Gentra Puregene Buccal Cell Kit, Qiagen, Valencia, CA, USA) with slight modifications, published elsewhere (17).

### TaqMan PCR assay

Allelic discrimination was performed by polymerase chain recation (PCR)-based *TaqMan* assays in the presence of two different fluorescently labeled probes, which allow for the detection of both alleles in a single reaction (Applied Biosystems Inc, Foster City, CA, USA): rs12913832 (*HERC2*), rs1545397 (*OCA2*), rs1426654 (*SLC24A5*), rs16891982 (*SLC45A2*), rs885479 (*MC1R*), rs6119471 (*ASIP*), rs12203592 (*IRF4*), and rs12896399 (*SLC24A4*) using optimized PCR-conditions [Volume 25μL; 10min 95°C, 50 cycles: 60sec 60°C, 15sec 92°C, performed on RotorGene 6000 (Qiagen, Valencia, CA)] (17).

### Data analysis

The 8-plex system, to predict eye and skin coloration, was developed by using a training set consisting of 803 samples. Two hundred and twelve additional newly-collected samples were used to test the developed 8-plex system. Statistical significance (*P* value) was calculated by using χ^2^ test, available at *vassarstats.net* (26). An error was defined as a predicted eye or skin color that did not match the binned actual eye or skin color. The significance of the increasing number of positive descriptions was calculated by using the binominal proportions statistical test, available at *http://www.fon.hum.uva.nl/Service/CGI-Inline/HTML/Statistics.html*, as well as the z-score at *vassarstats.net* (26) and the “z-score calculator” (*http://sampson.byu.edu/courses/z2p2z-calculator.html*). Further calculators were used from *statpages.org* (27).

## Results

### Selection of rs12896399 and sequence analysis of its flanking region

The SNP rs12896399 was selected based on its correlation with human eye coloration. This was shown in genome-wide association studies (11,12,15) and supported by a χ^2^ test using the training set (803 samples). However, from this sample set only samples from European descendants (n = 555) were used, which showed a relative even distribution among the three eye-color bins: blue (n = 195), green (n = 170), and brown (n = 190). Our data confirmed the significant correlation of rs12896399 with human eye color (χ^2^_4_: 14.09, *P* value: 0.007, Cramer’s V = 0.1127). However, a quantifying agreement test considered the strength as “poor” (κ = 0.063). Based on these results, rs12896399 was used in a newly added second step of the eye-color prediction.

Rs12896399 is located 15 262bp upstream of *SLC24A4* on chromosome 14. Sequence analysis of the flanking regions, 500bp up- and downstream of rs12896399 (1,001bp), revealed that this section is highly conserved within primates. The sequence identities were 99% for *pan troglodytes* (chimpanzee) and *pan paniscus* (bonobo), 98% for *nomascus leucogenys* (gibbon), 97% for *pongo abelii* (orang-utan), and 94% *macaca muletta* (rhesus macaque).

Further investigation using TFSEARCH (28) detected a possible binding site for transcription factor AML-1a with a score of 87.4 (percentage match). The binding site is specific for the ancestral G-allele. The transcription factors, AML-1a and AML-1b, are proteins from the same gene, *RUNX1*, produced by alternative splicing (29). Both forms have a DNA-binding domain. AML1 can improve its DNA binding capacity by forming a complex with a non-DNA binding protein (CBF beta) (30). AML1 recognizes the DNA sequence TGPyG**G**T, where Py stands for pyrimidine (T or C) (30). The bold **G** indicates the position of rs12896399. All positions of the flanking sequence match the binding site of AML1 except Py. At this position is a G, thereby reducing the score to 87.4. This analysis shows that the sequence around rs12896399 may be important and could contain regulatory regions.

### Eye and skin color prediction based on 8 SNPs

**Figure 1:**
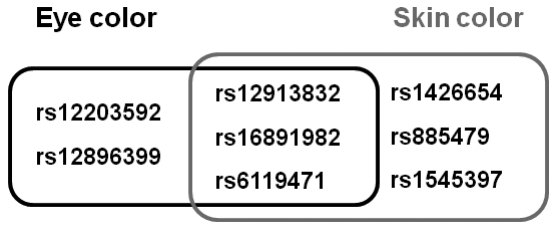
Single nucleotide polymorphisms used for eye- and skin-color prediction based on the 8-plex system: rs12203592, rs12896399, rs12913832, rs16891982, and rs6119471 are used to predict eye color; rs12913832, rs16891982, rs6119471, rs1426654, rs885479, and rs1545397 are used to predict skin coloration.

The 7-plex system to predict eye and skin coloration (17) was improved by adding one SNP (rs12896399) and by changing the instructions on how to interpret the genotypes unambiguously. Five of the eight SNPs are used to predict eye color (blue, green, brown, not blue, and not brown) and six to predict skin coloration (light, not dark and not light), whereby three are shared (Figure 1). The newly-designed 8-plex system was established on 803 samples (training set) (Table 1).

**Table 1:** Eye-color prediction: outcome for training set (n = 803)

Population*	Eye color bin	Eye color predicted (1)	Error	Further prediction (2)	Error
AA (43)	brown (43)	brown (31)	0		
		not blue (12)	0	brown (8)	0
				not blue (4)	0
SA (27)	brown (27)	brown (21)	0		
		not blue (6)	0		
EA (35)	brown (35)	brown (34)	0		
		not blue (1)	0		
E (555)	blue (195)	blue (11)	0		
		green (2)	2		
		not brown (175)	0	blue (37)	0
				not brown (138)	0
		not blue (7)	7		
	green (170)	green (1)	0		
		blue (1)	1		
		not blue (92)	0	brown (5)	5
				not blue (87)	0
		not brown (76)	0	blue (6)	6
				not brown (70)	0
	brown (190)	brown (2)	0		
		not blue (187)	0	brown (24)	0
				not blue (163)	0
		not brown (1)	1		
mix (143)	blue (11)	not brown (10)	0	blue (2)	0
				not brown (8)	0
		not blue (1)	1		
	green (20)	green (2)	0		
		brown (1)	1		
		not blue (10)	0		
		not brown (7)	0	blue (1)	1
				not brown (6)	0
	brown (112)	brown (45)	0		
		not blue (67)	0	brown (14)	0
				not blue (53)	0
**total (803)**			**13**		**12**
					

*AA – African-American, SA – South Asian, EA – East Asian, E –European.

**Figure 2:**
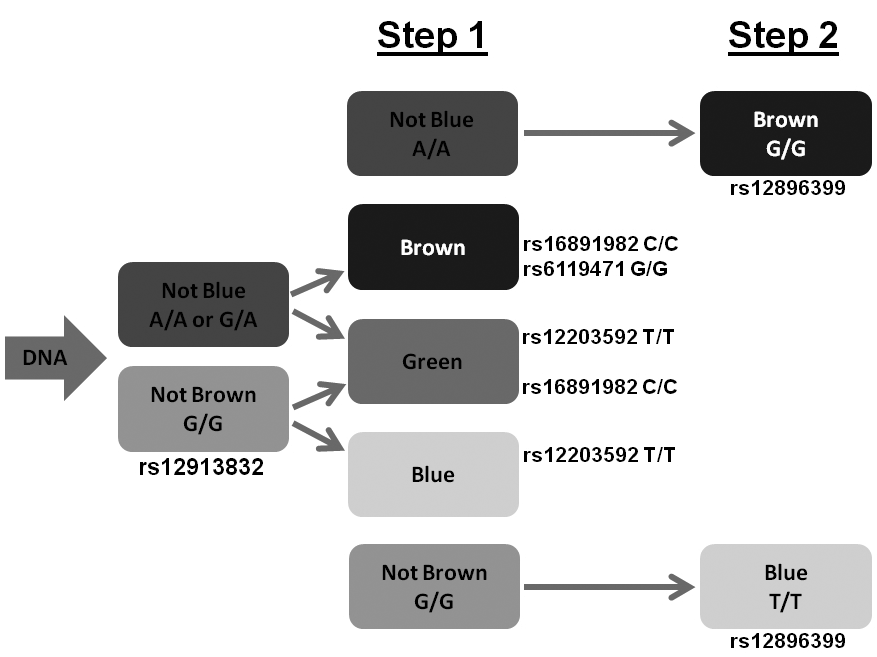
Schematic representation of the eye-color predictor (8-plex system): Step 1: eye color is predicted of being not brown (ie, green or blue) or not blue (ie, brown or green). Eye color can be further refined as being brown, green, or blue, depending on homozygous genotypes of rs16891982, rs6119471, and rs12203592. Step 2: follows only if step 1 results in “not brown” or “not blue.” Brown eye color is predicted by A/A at rs12913832 and G/G at rs12896399. Blue eye color is predicted by G/G at rs12913832 and T/T at 12896399.

The prediction for eye color is a two-step procedure: First, rs12913832 (*HERC2*) distinguishes eye colors as being not blue (ie, brown or green) or not brown (ie, green or blue). Further distinctions for predicting eye color as being brown, green, or blue are dependent on the genotypes of three additional SNPs: rs12203592, rs16891982, and rs6119471 (Figure 2). Brown eye color is predicted by the following genotype combinations: A/A or G/A at rs12913832, plus either G/G at rs6119471, or C/C at rs16891982; green eye color is predicted by G/G at rs12913832 plus C/C at rs16891982, or by G/A at rs12913832 plus T/T at rs12203592; and blue eye color is predicted by G/G at rs12913832 plus T/T at rs12203592.

The second step proceeds with samples that were not positively described as being brown, green, or blue in the first step (Figure 2). Samples that are homozygous for G/G at rs12913832 (ie, not brown) plus T/T at rs12896399 are then predicted to have blue eyes, while samples that are homozygous for A/A at rs12913832 (ie, not blue) plus G/G at rs12896399 are predicted to have brown eyes. This step utilizes the newly-included SNP rs12896399 (*SLC24A4*) and leads to increased numbers of positively described eye colors.

Table 1 shows the outcome for 803 samples from various populations, including African-American, South Asian, East Asian, European descendants, and mixed populations (training set). Thirteen errors were found within the first step of eye-color prediction; eleven of them were in the European population. Ten of the thirteen errors arose from rs12913832, which is the primary SNP on which the prediction relies (Figure 2) (24). The error-rate calculation is based on the European population since they show a wide eye-color variation. Including other populations in this calculation may introduce a bias and may lead to lower error rates by increasing the sample size but not increasing the probability of having blue, green, or brown eye colors. The error rate for the first step was calculated at 2%, which is comparable to the 7-plex system (3%) (24).

Out of 803 samples, the second step of the eye color prediction increased the number of positive descriptions by 97 samples (151 + 97 = 248). This increase equals approximately 12% for all samples. Of these predictions, twelve were erroneous (Table 1). These errors occurred for green-eyed individuals that were predicted to have either brown or blue eye color. The error rate for the second step was also calculated at 2% for the European population.

The skin color prediction of the 8-plex system utilizes six SNPs (Figure 1). In a similar manner to the eye color, the skin color is binned into three groups: light, medium, and dark. The skin-color predictor utilizes five SNPs to predict a lighter skin color and one to predict darker shades. Skin coloration is predicted in an elimination process. Non-dark skin color (ie, light or medium) is predicted by any two of the following alleles: G/G at rs12913832, G/G at rs16891982, A/A at rs1426654, T/T at rs1545397, or A/A at rs885479. Light skin color is predicted by more stringent conditions: G/G at rs12913832, plus G/G at rs16891982, and A/A at rs1426654. Non-light skin color (ie, medium or dark) is predicted by G/G at rs6119471.

Table 3 shows the outcome for skin color of the 803 training samples. Since this predictor utilizes only homozygous genotypes, the outcome can be inconclusive (ie, no prediction could be made). Of 803 samples tested, 600 (75%) predictions were made, resulting in only 4 errors (1%). The four erroneous predictions described skin color as being non-dark for dark-skinned individuals. The positive predictions for light skin coloration were all correct (255 of 600 predictions).

**Table 3:** Skin-color prediction

Outcome for training set (n = 803)					Outcome for test set (n = 212)			
Population*	Skin color bin	Skin color predicted	Error	Inconclusive	Skin color bin	Skin color predicted	Error	Inconclusive
**AA**	dark (39)	not light (15)	0	24	dark (8)	not light (4)	0	4
	medium (4)	not light (1)	0	3				
**SA**	dark (5)	not dark (1)	1	4	dark (1)			1
	medium (21)	not dark (2)	0	19	medium (7)			7
	light (1)			1	light (1)			1
**EA**	light (30)	not dark (9)	0	21	light (10)	not dark (1)	0	9
	medium (5)	not dark (1)	0	4				
**E**	light (555)	light (244)	0	43	light (114)	light (60)	0	7
		not dark (268)	0			not dark (47)	0	
**mix**	dark (22)	not light (5)	0	14	dark (5)	not light (1)	0	4
		not dark (3)	3					
	medium (48)	not dark (7)	0	37	medium (18)	not dark (4)	0	14
		not light (4)	0					
	light (73)	light (11)	0	33	light (48)	light (3)	0	34
		not dark (29)	0			not dark (11)	0	
	total (803)	600	4	203	total (212)	**131**	**0**	**81**
		75%	1%	25%		62%		38%

*AA – African-American, SA – South Asian, EA – East Asian, E –European.

### Verification of the 8-plex system with 212 additional samples

212 samples were collected to test the newly-developed 8-plex system. Eight of these newly collected samples were associated with African-American, nine with South Asian, ten with East Asian, 114 with European, and 71 with mixed populations.

The eye-color prediction followed a two-step procedure (Table 2), whereby the first step led to 56 positive descriptions, in which 2 errors occurred (error rate of 1.75%). The second step, including rs12896399, led to 30 further positive descriptions (56 + 30 = 86). Three errors were found in the second step (error rate equals 2.73%). All together five errors were counted; two arose from rs12913832, which was recently identified within the 7-plex system as an error source that led to a 3% error rate (24). The other three errors were related to the newly included rs12896399. All five errors found in the test set (n = 212) of the 8-plex system occurred in the European population and led to an error rate of approximately 5% (4.54%).

**Table 2:** Eye-color prediction: outcome for test set (n = 212)

Population*	Eye color bin	Eye color predicted (1)	Error	Further prediction (2)	Error
AA (8)	brown (8)	brown (7)	0		
		not blue (1)	0	brown (1)	0
SA (9)	green (1)	green (1)	0		
	brown (8)	brown (7)	0		
		not blue (1)	0	brown (1)	0
EA (10)	brown (10)	brown (10)	0		
E (114)	blue (51)	blue (3)	0		
		not brown (46)	0	blue (10)	0
				not brown (36)	0
		not blue (2)	2		
	green (38)	green (1)	0		
		not blue (21)	0	brown (2)	2
				not blue (19)	0
		not brown (16)	0	blue (1)	1
				not brown (15)	0
	brown (25)	not blue (25)	0	brown (3)	0
				not blue (22)	0
mix (71)	blue (4)	blue (1)	0		
		not brown (3)	0		
	green(8)	green (2)	0		
		not blue (6)	0		
	brown (59)	brown (24)	0		
		not blue (35)	0	brown (12)	0
				not blue (23)	0
total (212)			2		**3**

*AA – African-American, SA – South Asian, EA – East Asian, E –European.

Table 3 shows the results for the skin-color predictor. A prediction could be made for 131 (62%) samples out of 212 samples. Of these, 63 were predicted to have light skin color and all of those were correct.

Taken together, the outcomes from the training and test sets are in concordance. The newly-developed eye- and skin-color predictor utilizing 8 SNPs is an improved version of the 7-plex system, leading to more precise descriptions including blue-eye and light-skin coloration. In addition, the new 8-plex system increased the number of positive descriptions significantly (*P* = 6.98e-15, z-score: -7.786) while keeping the error rate low.

## Discussion

The newly developed 8-plex system demonstrated improved results compared with the former 7-plex system (17) by its ability to predict blue eye and light skin color. The number of positive descriptions for eye color increased significantly (*P* = 6.98e-15, z-score: -7.786), while keeping a low error-rate at approximately 5%.

In more detail, the 7-plex system utilizes seven SNPs (rs12913832, rs16891982, rs6119471, rs1426654, rs885479, rs1545397, and rs12203592) from seven genes. Six of them (rs12913832, rs16891982, rs6119471, rs885479, rs1545397, and rs12203592) are used to describe eye color as being brown, green, not brown, or not blue. All seven SNPs are used to predict skin coloration as being not light or not dark (17). The new 8-plex system utilizes 8 SNPs including all from the 7-plex system plus rs12896399. The newly-added SNP is located 15kb upstream of *SLC24A4*, a gene coding for a sodium/potassium/calcium exchanger (31). Further analysis of the non-coding region flanking this SNP revealed a high degree of conservation among primates, which could lead to the assumption that rs12896399 may be located in a gene-regulatory region. The gene product of *SLC24A4* is expressed in different tissues such as the brain, aorta, lung, and thymus as well as many others (31); its function in pigmentation needs further elucidation (32). However, several GWAS found that rs12896399 is associated with eye, hair, and skin pigmentation (10-12,15,16). We confirmed that rs12896399 was significantly correlated (*P* = 0.007) with brown, green, and blue eye colors, but the strength was poor (κ = 0.063).

The 8-plex eye-color predictor, based on five SNPs (rs12913832, rs16891982, rs6119471, rs12203592, and rs12896399), uses rs12203592 to predict blue eye color, and the introduction of rs12896399 increases the number of positive predictions significantly, but leads only to brown and blue eye color predictions. The error rate for the eye-color prediction was estimated to be 5%, which is slightly higher than the 7-plex system with 3% (24) but within the same range. The 8-plex system consists of two steps, each of which contributes to the error rate, approximately 2% for the first step and 3% for the second step. Most errors occurred in the second step for green-eyed individuals who were predicted to have either blue or brown eye colors. Increasing the number of positive calls may increase the error rate. It was considered to revert to simplified categories such as “light” and “dark” eyes to increase accuracy (33). On the other hand, further research of additional genes and polymorphisms may result in more precisely defined eye-color outcomes (34).

Six SNPs (rs12913832, rs16891982, rs6119471, rs1426654, rs885479, and rs1545397) are used to predict skin coloration as being light, not dark, or not light. Skin-color prediction depends on homozygous genotypes; therefore, inconclusive results are possible. The call rate was highest for the European population, confirming earlier results (24). The percentage of Europeans in the training and test sets differed; they were approximately 70% and 54%, respectively. Therefore, it was expected that the call rate for all samples was lower in the test set. However, the call rate for the European population within the test set was approximately 94%. It should be noted that no errors occurred for skin-color predictions within the test set. More research in this field will increase the number of positive and accurate predictions.

A missing persons report typically contains general descriptive information about an individual. With skeletonized human remains, physical characteristics such as age, ethnicity, sex, and height can often be determined by forensic anthropologists. While these metrics are extremely useful, it is not possible to determine eye, hair, and skin color from skeletal remains by existing methods. The utilization of DNA analysis to predict visible pigment-related features of unidentified human remains will enhance current efforts to gather obtainable identifying information from a decedent. It would be beneficial for forensic scientists to design and validate a multiplex SNP-assay that would target these eight SNPs. Regarding equipment, the method of choice for many forensic laboratories could be a multiplex-PCR followed by a single-base primer extension reaction, creating fluorescently labeled oligonucleotides of distinct lengths. Multicolor capillary electrophoresis could be used for detection. This method would make efficient use of genomic DNA, could be applied on degraded DNA, and is inexpensive.
